# Determinants of Children's Exhaled Nitric Oxide: New Insights from Quantile Regression

**DOI:** 10.1371/journal.pone.0130505

**Published:** 2015-07-27

**Authors:** Yue Zhang, Kiros Berhane, Sandrah P. Eckel, Muhammad T. Salam, William S. Linn, Edward B. Rappaport, Theresa M. Bastain, Frank D. Gilliland

**Affiliations:** 1 Department of Internal Medicine, University of Utah, Salt Lake City, Utah, United States of America; 2 Department of Family and Preventive Medicine, University of Utah, Salt Lake City, Utah, United States of America; 3 Veteran Affairs Salt Lake City Health Care System, Salt Lake City, Utah, United States of America; 4 Department of Preventive Medicine, University of Southern California, Los Angeles, California, United States of America; 5 Department of Psychiatry, Kern Medical Center, Bakersfield, California, United States of America; University of Athens, GREECE

## Abstract

While the fractional concentration of exhaled nitric oxide (FeNO) has proven useful in asthma research, its exact role in clinical care remains unclear, in part due to unexplained inter-subject heterogeneity. In this study, we assessed the hypothesis that the effects of determinants of the fractional concentration of exhaled nitric oxide (FeNO) vary with differing levels of FeNO. In a population-based cohort of 1542 school children aged 12–15 from the Southern California Children's Health Study, we used quantile regression to investigate if the relationships of asthma, socio-demographic and clinical covariates with FeNO vary across its distribution. Differences in FeNO between children with and without asthma increased steeply as FeNO increased (Estimated asthma effects (in *ppb*) at selected 20^th^, 50^th^ and 80^th^ percentiles of FeNO are 2.4, 6.3 and 22.2, respectively) but the difference was steeper with increasing FeNO in boys and in children with active rhinitis (p-values<0.01). Active rhinitis also showed significantly larger effects on FeNO at higher concentrations of FeNO (Estimated active rhinitis effects (in *ppb*) at selected 20^th^, 50^th^ and 80^th^ percentiles of FeNO are 2.1, 5.7 and 14.3, respectively). Boys and children of Asian descent had higher FeNO than girls and non-Hispanic whites; these differences were significantly larger in those with higher FeNO (p-values<0.01). In summary, application of quantile regression techniques provides new insights into the determinants of FeNO showing substantially varying effects in those with high versus low concentrations.

## Introduction

Due to the great potential of fractional exhaled nitric oxide (FeNO) as a noninvasive marker of eosinophilic airway inflammation [1–8], extensive research has been undertaken in recent years to explore the important host and environmental factors as determinants of FeNO. Kovesi et al found FeNO concentration among healthy school children to be significantly associated with race, age and height [9]. Levesque et al found that gender, serum total immunoglobulin E (IgE) levels and current upper respiratory tract infection symptoms significantly contributed to the FeNO variability in healthy nonsmoking African Americans [10]. Olivieri et al and Taylor et al recommended that gender should be considered in creating FeNO reference values even when atopy, smoking, asthma symptoms and steroid usage have been taken into account [11, 12]. Yao et al determined that the upper limits of normal FeNO in Asian children are higher than in Caucasian children [13]. Van der Valk et al identified FeNO in children was associated with 3 genetic variants [14].

To date, most studies have investigated the determinants of FeNO using conventional mean regression techniques. This method assumes that the effects of predictors of FeNO have a constant relationship across the distribution of FeNO from low to high concentrations. However, this assumption may obscure potentially important variation in effects by averaging the effects of a predictor across the distribution of FeNO. For example, the same level of exposure may cause a relatively larger response in subjects whose FeNO is already high, but no response in subjects with low-to-moderate prior FeNO levels. To the best of our knowledge, no studies have investigated heterogeneity in effects of FeNO determinants amongst individuals with underlying high versus low FeNO levels.

To investigate heterogeneity in effects of determinants across the distribution of FeNO concentrations, we applied a quantile regression (QR) approach. The QR approach [15] provides a useful framework to assess the effects of risk factors that could vary across the distribution of FeNO, providing a more complete picture of covariate effects on FeNO. The QR approach also has the additional advantage of being independent on the normality assumption inherent in regular mean regression. While our analysis of FeNO data using QR is novel, its advantages have already provided new insights in pulmonary function research [16,17].

The present study aimed to investigate heterogeneity in the relationships of important determinants of FeNO across different percentiles of FeNO among school children who participated in the Southern California Children's Health Study (CHS). We focus on the school children in this study because they suffer greater burden from respiratory disease, such as asthma, than adults.

## Methods

### Study Population

Study participants were drawn from an ongoing, population-based CHS cohort of children who were recruited from kindergarten or first grade classrooms in 2002–2003 from 13 southern California communities. The research protocol was approved and documented by the University of Southern California Health Sciences Campus Institutional Review Board. Written informed consent was obtained from a parent or guardian on behalf of each child participant. More information on the study design of this cohort has been reported elsewhere [18].

### FeNO measurement

Details of the FeNO collection and quality control approaches have been reported elsewhere [19]. Briefly, FeNO measurements were collected at schools from March to June 2010 using an online breath collection technique. Online measurements were performed at 50ml/s expiratory flow rates using EcoMedics CLD-88-SP analyzers, with DeNOx accessories to provide NO-free inhaled air (EcoPhysics Inc., Ann Arbor, MI, USA/Duernten, Switzerland), according to American Thoracic Society (ATS) guidelines [20]. Each child provided three maneuvers recorded at the 50 ml/s target flow rate. Additional maneuvers may have been recorded if the technician judged it necessary to replace one that was unsatisfactory. Plateau acceptability required < = 10% NO concentration variability [20]. Plateau concentration for each maneuver was taken from the 3 second plateau with the minimum NO coefficient of variation. FeNO was represented by the mean of two or three ATS acceptable plateaus that differed by less than 15% or 1 *ppb* where all ATS acceptable plateaus were less than 10 *ppb*. In the present analysis, acceptable FeNO was measured in 1542 subjects on March through June of 2010 school year of the study.

### Covariate Information

We collected covariate data from questionnaires completed by parents/guardians, including socio-demographic factors such as child's gender, race/ethnicity, date of birth, and community of residence. Children were classified as having asthma if the parent/guardian reported that a doctor had "ever diagnosed the child as having asthma." Children who enrolled were asked to fill out a questionnaire regarding their health information in the past 12 months at the time of FeNO measurement [18,21]. Information on current rhinitis was defined based on children's answers to two nested questions ("In the past 12 months, have you had a problem with sneezing, or a runny or blocked nose when you did not have a cold or the flu?" and "In just the last 30 days, when did you have this nose problem?") with categories of 1) No rhinitis in last 12 month, 2) rhinitis between 1 and 12 months ago, 3) rhinitis between 7 days and 1 month ago and 4) rhinitis within last 7 days, hereafter also referred to as active rhinitis. BMI was calculated based on height and weight measurement made on the day of FeNO measurement. Overweight and obesity status were determined based on age and gender specific cutoffs from CDC BMI-for-age growth charts (SAS program available at http://www.cdc.gov/nccdphp/dnpao/growthcharts/resources/sas.htm). Inhaled corticosteroid (ICS) use was defined based on children's answers to the questions about medication requirement for asthma and wheezing in the last 12 months and in just the past month, with categories of 1) Never use, 2) ICS use between 1 and 12 months ago, 3) ICS use between 7 days and 1 month ago, and 4) ICS use within 7 days.

### Statistical analysis

Descriptive and exploratory data analyses were conducted to examine the characteristics of the whole study population and by asthma status to characterize the distribution of the FeNO measurements. QR approach [15] was used to model the relationship between a set of covariates and specific percentiles (or quantiles, denoted by τ) of the response variable, FeNO, as shown below:

$$Q_{FeNO}\left(\tau \right)=\beta_{0}\left(\tau \right)+\beta_{1}\left(\tau \right)X_{i.}$$

In the QR model, the regression coefficients are indexed by quantile τ (where, for example, τ = 0.5 corresponds to the median) and quantify the change in the value of the quantile of FeNO, Q_FeNO_(τ), associated with a one unit change in the predictor variable X. In this study, QR was used to examine the mutually adjusted effects of asthma, socio-demographic and clinical factors on quantiles of FeNO. The list of these priori determinants of FeNO was selected based on existing literature and study design. All models were adjusted for the design variables age and community of residence as well as BMI percentiles. Interaction effects of asthma by active rhinitis or gender were also examined. Sensitivity analyses were conducted among children reporting no ICS use in last 12 months and children reporting not having both allergic and infectious rhinitis in last 30 days, respectively. We fitted the QR models at quantiles ranging from 0.10 to 0.90, with 0.01 increments. Since inference in quantile regression is independent of the normality assumption, log transformation of FeNO is not needed. All models were fitted using the *quantreg* package in R (a statistical software which is freely available at http://cran.r-project.org/) using method discussed in [15]. Pairwise Wald tests were used to test the equality of covariate effects across selected quantiles (0.2, 0.5 and 0.8). Statistical significance was assessed assuming a 0.05 significance level and a two-sided alternative hypothesis.

## Results

Participants of this study were between 12 and 15 years old and were equally divided between boys and girls (Table 1). The majority of the subjects were Hispanic (Primarily Mexican or Central American descent) (56.6%) and Non Hispanic White (31.3%). The geometric mean (SD) of FeNO for the cohort was 15.8 *ppb* (SD = 2.1). Consistent with previous literature, we found that gender and race/ethnicity were associated with FeNO (P-value: <0.0001 for gender and 0.003 for race/ethnicity), with boys and Asians, African Americans having significantly higher FeNO levels. Children with asthma had significantly higher FeNO level compared to children without asthma (p-value<0.0001). Almost 16% of the children had rhinitis within the last 7 days before the FeNO measurements. We found that more recent occurrence of rhinitis was associated with higher FeNO level (P-value<0.0001) while children with rhinitis within last 7 days have highest FeNO level (geometric mean 21.6 *ppb* with geometric standard deviation 2.4). Less than 10% of children had rhinitis between 7 days and 1 month prior to FeNO measurement, and about 6% had it between 1 month and 12 months prior. Most children in this population-based cohort did not have asthma (80% of the full cohort). Among children with asthma, slightly more than half were boys, almost a quarter had rhinitis within the last 7 days before FeNO measurements, and less than 10% had ICS use within 1 month preceding FeNO measurement. Both current rhinitis and male were significantly associated with FeNO measurement (P-value: 0.001 for current rhinitis and 0.02 for gender). More information about the distribution of FeNO in the study cohort by selected children's characteristics is shown in the Online Repository (S1 Table).

**Table 1:** Selected Characteristics of CHS FeNO Study Participants and Children by Asthma Status*.

	All Children			Children without Asthma			Children with Asthma		
	N**	FeNO Mean(SD)^$^	P-Value^†^	N**	FeNO Mean(SD)^$^	P-Value^†^	N**	FeNO Mean(SD)^$^	P-Value^†^
**Gender**	** **	** **							
Female	793(51.4%)	14.4(2)	<0.0001	657(53.1%)	13.4(1.9)	<0.0001	136(44.6%)	19.8(2.2)	0.031
Male	749(48.6%)	17.4(2.2)		580(46.9%)	15.7(2.1)		169(55.4%)	24.4(2.4)	
**Race/Ethnicity**									
Hispanic White	873(56.6%)	15.5(2.1)	0.003	711(57.5%)	14.2(2.1)	0.0003	162(53.1%)	22.8(2.3)	0.919
Non-Hispanic White	482(31.3%)	15.5(2)		381(30.8%)	14.3(1.9)		101(33.1%)	21.3(2.2)	
African American	27(1.8%)	19.4(2.1)		21(1.7%)	19.3(2)		6(2.0%)	19.8(2.5)	
Asian	64(4.2%)	22(2.2)		51(4.1%)	21.1(2.2)		13(4.3%)	25.7(2.3)	
Others	96(6.2%)	14.6(2.2)		73(5.9%)	12.9(1.9)		23(7.5%)	21.6(2.9)	
**Rhinitis in Last 12 Months**									
None	1031(66.9%)	14.4(2)	<0.0001	881(71.2%)	13.7(2)	<0.0001	150(49.2%)	19.2(2.2)	0.002
1–12 months ago	96(6.2%)	14.8(1.9)		71(5.7%)	13.4(1.8)		25(8.2%)	19.5(1.9)	
7 Days—1 month ago	152(9.9%)	18.0(2.2)		98(7.9%)	15.1(2.1)		54(17.7%)	24.8(2.3)	
Within last 7 Days	250(16.2%)	21.6(2.4)		175(14.1%)	19(2.2)		75(24.6%)	29.2(2.5)	
**Inhaled Corticosteroid Use in Last 12 Months** ^‡^									
Did not use	—-	—-		—-	—-		257(84.3%)	21(2.3)	0.007
Used 1–12 months ago	—-	—-		—-	—-		15(4.9%)	23.9(2.3)	
Used within last 1 month	—-	—-		—-	—-		28(9.2%)	35.1(2.4)	
**Total**	1542	15.8(2.1)		1237	14.5(2)		305	22.2(2.3)	

*: The participants in this study are children who were enrolled in the Southern California Children’s Health Study (CHS) and had FeNO measurements at 2009–2010 school year.

**: Numbers do not always sum to the total due to missing data.

^**†**^: Tests for the equality of group means of log-transformed FeNO (did not include missing category).

^**‡**^: The distribution of Inhaled Corticosteroid Use in Last 12 Months is only considered in the sub-cohort of children with asthma, since only children with asthma received the medication.

^**$**^: Geometric mean and geometric standard deviation.

Table 2 presents the estimated differences in the selected quantiles of FeNO (*ppb*) associated with asthma, rhinitis in the last 12 months, gender and race/ethnicity. We found that the effects of asthma on FeNO steeply increased in magnitude across the distribution of FeNO. In other words, there was substantial heterogeneity of the effect of asthma as asthma tended to have a larger effect on the FeNO level as the FeNO level increased. The estimated FeNO level at 20^th^ percentile for children with asthma was 2.4 *ppb* (p-value<0.01) higher than that for children without asthma. In contrast, the estimated FeNO level at 80^th^ percentile for children with asthma increased by 22.2 *ppb* (p-value<0.01). The estimated increase in the 80^th^ percentile of FeNO associated with asthma was about 10 times, 4 times and 2 times larger than children without asthma at 20^th^, 50^th^ and 60^th^ percentiles, respectively. These pairwise differences of the effect of asthma among those with low levels (quantile = 0.2), intermediate levels (quantile = 0.5) and high levels (quantile = 0.8) were all statistically significant with p-value <0.01 (see S2 Table in the online supplement). We found further evidence for a non-constant effects of rhinitis symptoms within 7 days before FeNO measurement, gender and Asian ethnicity. Especially, we discovered a substantial heterogeneity in the impact of rhinitis within 7 days before FeNO testing on the distribution of FeNO. While the estimated FeNO level at 20^th^ percentile for children with active rhinitis within 7 days before FeNO testing was only 2.1 *ppb* (p-value<0.01) higher than children without active rhinitis, the estimated FeNO level at 80^th^ percentile for children with active rhinitis is 14.3 *ppb* (p-value<0.01) higher.

**Table 2:** Estimated Differences (Standard Error) in FeNO associated with Asthma, Rhinitis, Gender and Race/Ethnicity at selected Quantiles of FeNO (ppb) ^†^.

	Selected Quantiles of FeNO				
	0.2	0.4	0.5 (median)	0.6	0.8
	Estimate (SE)	Estimate (SE)	Estimate (SE)	Estimate (SE)	Estimate (SE)
**Intercept** ^‡^	6.8(0.9)**	8.6(1.0)**	10.7(1.7)**	13.5(1.9)**	27.9(6.9)**
**Asthma**					
No	—-	—-	—-	—-	—-
Yes	2.4(0.5)**	4.2(1.0)**	6.3(1.4)**	10.1(2.1)**	22.2(3.2)**
**Rhinitis in Last 12 Months**					
None	—-	—-	—-	—-	—-
1–12 months ago	0.8(0.6)	-0.2(0.7)	0.0(0.9)	-0.3(1.2)	-0.4(2.5)
7 Days—1 month ago	0.8(0.5)	1.7(0.7)**	1.6(0.9)*	1.8(1.2)	3.7(2.2)*
Within last 7 Days	2.1(0.5)**	3.6(1.1)**	5.7(1.5)**	7.5(1.6)**	14.3(3.5)**
**Gender**					
Female	—-	—-	—-	—-	—-
Male	0.9(0.3)**	1.5(0.4)**	2.1(0.5)**	2.4(0.8)**	7.2(1.5)**
**Race/Ethnicity**					
Hispanic White	—-	—-	—-	—-	—-
Non-Hispanic White	-0.1(0.4)	-0.5(0.5)	-0.3(0.6)	-0.7(0.8)	-2.5(1.6)*
African American	3.7(0.6)**	3.8(2.0)*	3.6(1.2)**	3.2(3.8)	6.3(7.9)
Asian	-0.4(1.1)	5.7(4.2)	10.8(2.5)**	9.8(3.3)**	9.6(1.8)**
Others	-1.2(0.7)	-0.9(0.7)	-0.9(1.4)	0.1(1.6)	-2.8(4.3)

*: P-value<0.05

**: P-value<0.01

^†^: Age, BMI percentile and community are adjusted for all above models.

^**‡**^: The intercept represents the estimated quantile of FeNO (in ppb) for children in the reference group of all covariates included in the model (e.g., 10.1 ppb is the estimated median FeNO for non-hispanic females without asthma and without rhinitis in the past year in the reference community at an average age (13) and BMI percentile (0.5).

**Fig 1:**
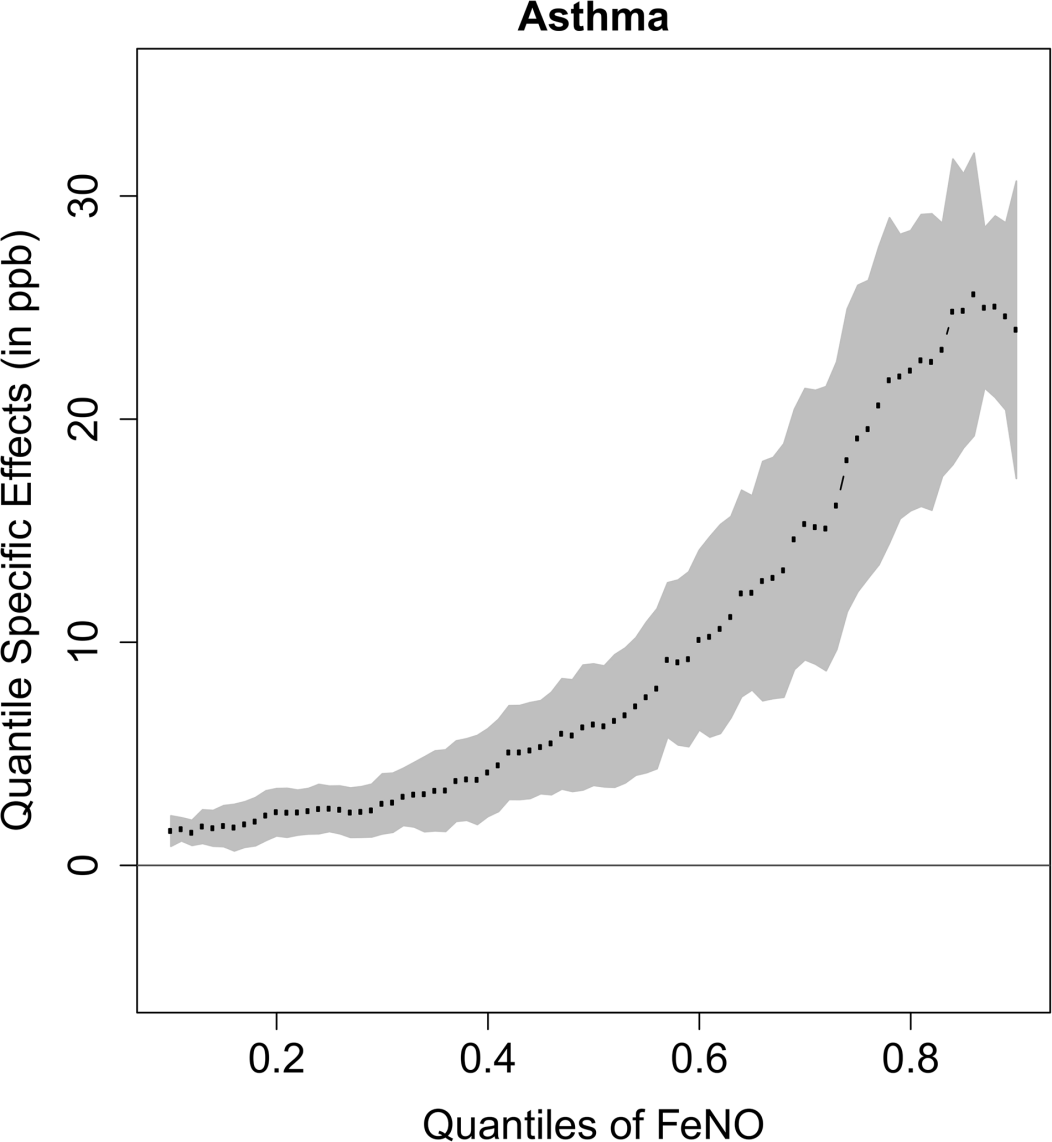
Asthma Effects on Different Quantiles of FeNO. The solid line shows the location of no covariate effect. The shaded gray area depicts a 95% pointwise confidence band for the quantile regression estimates, which is represented by the dot line.

**Fig 2:**
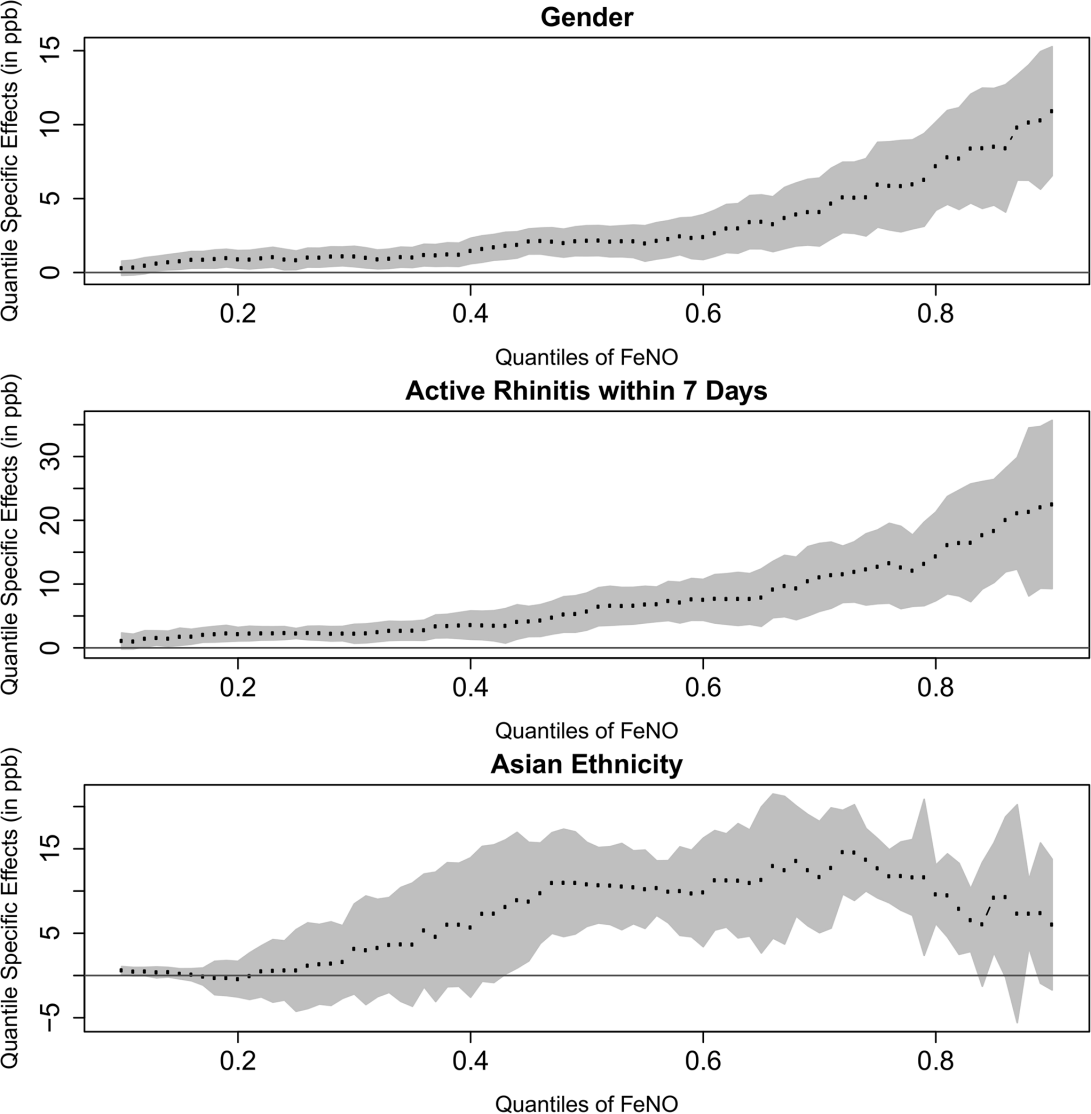
Effects of Gender (Reference group: Girls Group), Asian (Reference group: Hispanic White Group)and Active rhinitis within last 7 days (Reference group: Non-Rhinitis Group) on Different Quantiles of FeNO.

Fig 1 shows the heterogeneity of the magnitude of effect of asthma on FeNO in a more complete way by presenting the estimated effects at quantiles ranging from 0.10 to 0.90. The figure shows that the differences in the effect associated with asthma were statistically significant across the distribution of FeNO but varied in magnitude from low to high FeNO concentrations. Although the effects of asthma were small in the lower tail of the distribution, the magnitude increased dramatically in the upper tail of the distribution. Similar patterns for the non-constant and increasing effects of active rhinitis, gender and race/ethnicity are shown in Fig 2.

**Fig 3:**
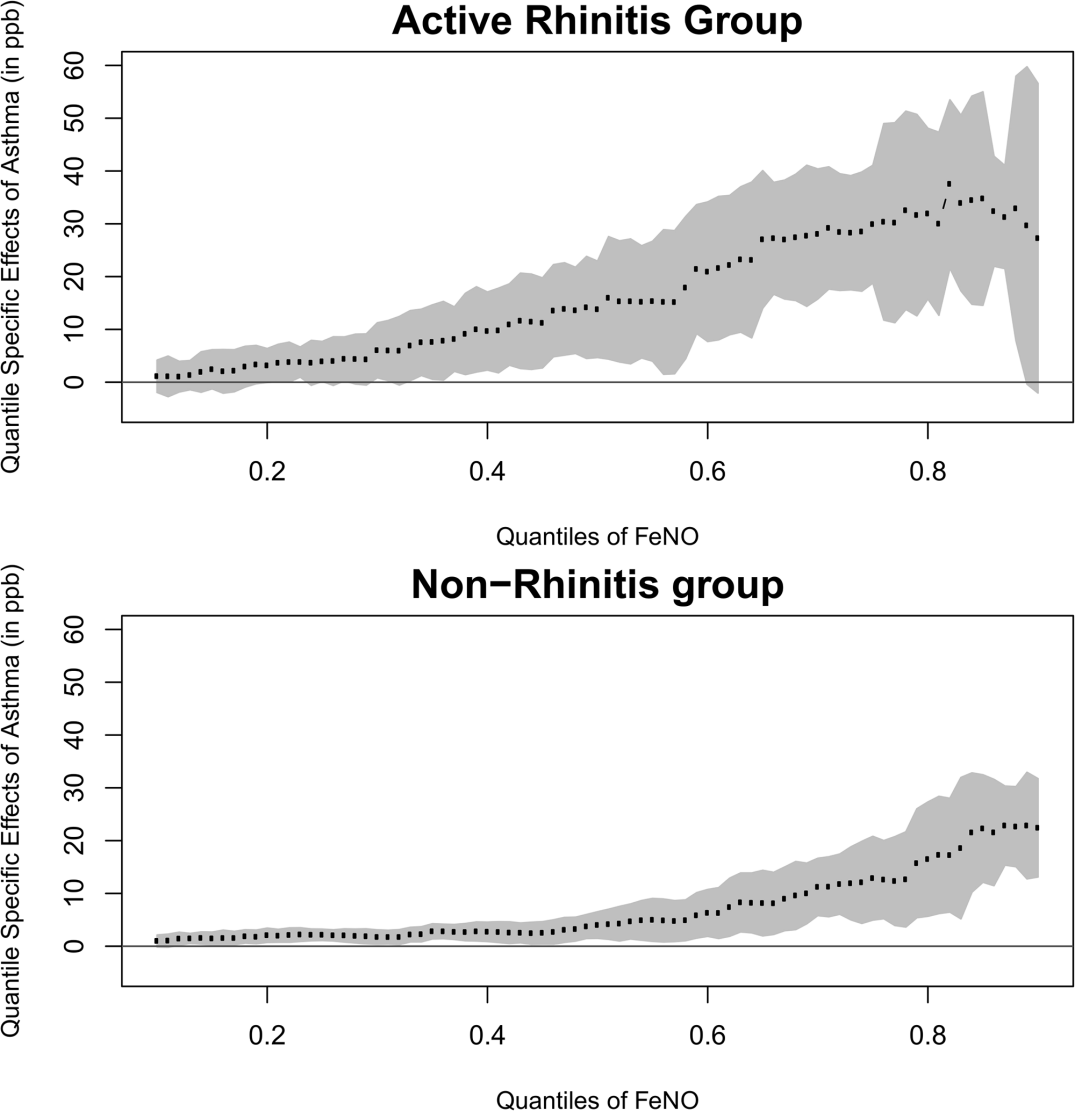
Current Rhinitis Specific Asthma Effects on Different Quantiles of FeNO (Reference Group: Non-Rhinitis Group).

**Fig 4:**
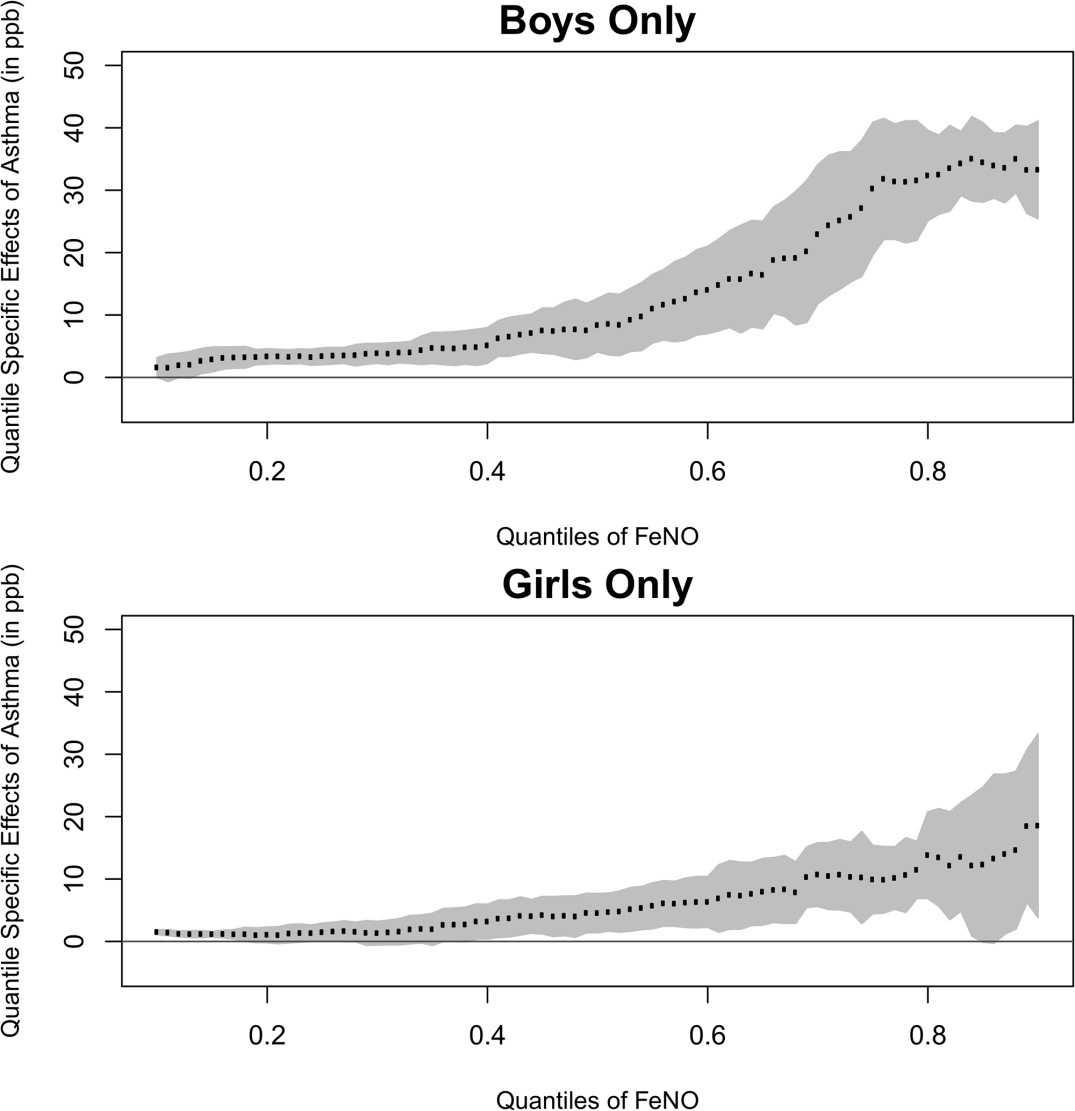
Gender Specific Asthma Effects on Different Quantiles of FeNO (Reference Group: Girls Group).

Table 3 presents estimates of FeNO differences associated with asthma by current rhinitis and gender. The pattern of steeply increasing effect of asthma with increasing FeNO was evident in both sexes and each rhinitis symptom group. At each quantile, asthma had consistently larger effects in boys than in girls and with increasing effects in the more recent occurrence of rhinitis symptoms. The differences of asthma effects between boys and girls were statistically significant only at the extreme upper tail of the FeNO distribution (interaction p-value = 0.002 at 0.8). Asthma had consistently larger effects in children with rhinitis symptoms within last 7 days than in children without rhinitis across the various quantiles. Figs 3 and 4 presents a summary of estimated effect of asthma by gender and current rhinitis groups, respectively, across quantiles ranging from 0.10 to 0.90. In both groups, the magnitude differences of FeNO concentrations associated with asthma was smaller in the lower tail of the FeNO distribution, but was considerably larger in the upper tail. The effect of asthma increased steeply as the percentile of interest increased. The rate of increase (effect size vs. percentile) was larger in boys than in girls. A similar pattern was also observed in the comparison between the active rhinitis group and the group without rhinitis symptoms. The race/ethnicity differences of the pattern of increasing asthma effects were not statistically significant (data not shown). The relationship of history of rhinitis symptoms on FeNO across its distribution did not significantly vary by gender and race/ethnicity (data not shown).

**Table 3:** Estimated differences (Standard Error) in FeNO associated with Asthma at Selected Quantiles of FeNO (ppb) by Categories of Rhinitis and Gender^†^.

	Selected Quantiles of FeNO				
	0.2	0.4	0.5 (median)	0.6	0.8
	Estimate (SE)	Estimate (SE)	Estimate (SE)	Estimate (SE)	Estimate (SE)
**Rhinitis in Last 12 Months**					
None	2.1(0.7)**	2.7(1.0)**	4.0(1.3)**	6.3(2.3)**	16.5(5.5)**
1–12 months ago	2.1(1.5)	3.6(1.3) **	6.4(3.2)**	5.0(3.6)	7.4(17.9)
7 Days—1 month ago	1.8(1.4)	7.2(2.1)**	11.0(3.5)**	13.5(4.5)**	27.9(4.5)**
Within last 7 Days	3.2(1.6)**	9.7(3.8)**	13.8(4.7)**	20.9(6.7)**	31.9(8.2)**
**Gender**					
Female	1.1(0.6) *	3.2(1.4)**	4.5(1.6)**	6.3(2.1)**	13.8(3.5)**
Male	3.4(0.6)**	5.1(1.5)**	8.4(2.2)**	14.0(3.6)**	32.4(3.7)**

*: P-value<0.05

**: P-value<0.01

^†^: Age, BMI percentile and community are adjusted for all above models.

Several models were fitted to assess the sensitivity of the results. Excluding subjects reporting ICS use in the past 12 months (N = 43) did not alter the significant effects and increasing trends in the magnitude of effects these determinants, and did not reveal any significant evidence of heterogeneity of effects for any of the other covariates, such as physical activity, BMI and community (data not shown). The analysis excluding subjects with both allergic and infectious rhinitis in the last 1 month (N = 79) showed that the results of non-constant effects of rhinitis symptoms within 7 days before FeNO measurement are very robust (data not shown).

## Discussion

Our results provide new insight into the heterogeneity of relationships of asthma, rhinitis, gender and race/ethnicity with FeNO in children. By allowing the effects of determinants to vary depending on the level of FeNO, quantile regression models present a more comprehensive picture of the covariate effects across the entire FeNO distribution. As a result, we identified potentially important insight about variation in the effects of determinants of FeNO across its distribution. As in many previous studies, we found significant difference in FeNO between children with and without asthma. Importantly, these differences varied in magnitude across the FeNO distribution. The effects of asthma were larger at higher FeNO concentrations, especially in boys and in children with rhinitis symptoms within 7 days before FeNO testing. Active rhinitis showed significant larger effects on FeNO at higher concentrations of FeNO. Boys had higher FeNO than girls that increase across FeNO levels. Children of Asian descent had significantly larger increases in the upper tail of the FeNO distribution. These novel insights suggest that the magnitude of effects of several key determinants vary across the full distribution of FeNO concentrations observed in a population. These findings suggest that assessment of asthma status using FeNO may be enhanced by using classification algorithms that account for these heterogeneity of effects, and imply that in clinical care, there is no "one size fits all" cutoff for FeNO and that more personalized reference values for FeNO would be preferable.

Our results extend and complement previous findings. Xu et al found that asthma and allergic rhinitis could increase the FeNO levels in Chinese schoolchildren near 10 years of age [22]. Kalpaklioglu et al also found that rhinitis and asthma are responsible for increased FeNO, irrespective of atopy [23]. Malinovschi et al found that intermediate or high FeNO and blood eosinophil count (B-Eos) values were independently associated with having asthma, wheeze and asthma attacks [24]. We found that the effect of asthma is much stronger in children who already have high FeNO level, especially for boys and children with active rhinitis. Our study also extends the association between FeNO and rhinitis to a more diverse population using a population-based cohort of school children from southern California, increasing the generalizability of the previous finding. The effect of active rhinitis symptoms within last 7 days was not constant but monotonically increases with increasing percentiles of FeNO. This implies that interpretation of FeNO level needs to account for rhinitis symptoms.

The strengths of the current study include the large, population-based study of school children, online FeNO testing, rigorous FeNO data quality control, and a thorough investigation of the effects of determinants on FeNO using a cutting-edge statistical approach.

The results from our study should be interpreted in light of some limitations. The cross sectional nature of the analysis precludes us from assessing the effects on FeNO with adjustments for some long term health confounders and temporal effects. Our covariates were collected based on questionnaire information; this approach might potentially introduce recall bias and misclassification. However, the questionnaire items we employ are widely used in similar studies and treated as standardized core questions to estimate the prevalence of asthma status and current nose symptoms [25]. The relationship between current rhinitis symptoms and FeNO is biologically plausible, because allergic rhinitis is associated with poor asthma control in children [26]. In addition, Malinovschi et al showed that elevated exhaled nitric oxide predicted the onset and prevalence of rhinitis symptoms in an adolescent cohort [27]. The findings in this study may not be generalizable to a clinical population as the study population was pubescent children who were primarily non-Hispanic white and Hispanic children in southern California. Due to the small proportion of the children of Asian descent in study cohort, the robustness of the finding in the no-constant effects of Asian group on FeNO need be further evaluated. The mean FeNO of the children with asthma not receiving inhaled steroid is relatively low implying well controlled asthma of the majority of the asthmatic children of the study. It would be ideal that the information of the Asthma Control Test (ACT), which is not available in CHS cohort, could be evaluated. The lack of puberty information in the study cohort limits our ability to have further understanding of the observed heterogeneity in gender effect on the FeNO. Meanwhile, it is also plausible that hormonal factors may influence FeNO level and as such gender differences we observed could be explained by difference in hormonal milieu between boys and girls. Further studies are warranted to investigate the role of sex steroid hormones and puberty in FeNO.

In summary, application of quantile regression techniques provides new insights into the determinants of FeNO showing substantially varying effects in those with high versus low concentrations. Our findings provide evidence that risk factors associated with FeNO, have different effects across the range of FeNO in children. Incorporating this heterogeneity of effects in interpreting FeNO may result in significant improvement in applications of FeNO. Further research is warranted to explore the variation of determinants effects in other populations and on "extended" (multiple-flow) measurements of FeNO as well as the methods for applying quantile regression to clustered data in the setting of longitudinal studies.

## Supporting Information

S1 Table Distribution of FeNO by Selected Participant Characteristics. (DOCX)

S2 Table Pairwise Tests of the Equality of Covariate Effects across Selected Quantiles. (DOCX)
