# Supplementary material for: Determinants of Children's Exhaled Nitric Oxide: New Insights from Quantile Regression
Source: PLoS One. 2015 Jul 27;10(7):e0130505. doi: 10.1371/journal.pone.0130505 (PMC4516246; doi:10.1371/journal.pone.0130505)
Supplement: S1 Table — (DOCX) [file pone.0130505.s001.docx]

**S1 Table: Distribution of FeNO by Selected Participant Characteristics**

|  |  | **Selected Quantiles of FeNO** | | | |
| --- | --- | --- | --- | --- | --- |
|  | **Median (IQR)** | **0.2** | **0.4** | **0.6** | **0.8** |
| **Gender** |  |  |  |  |  |
| Female | 12.3 (12.7) | 8.3 | 11.0 | 15.0 | 24.7 |
| Male | 14.7 (17.4) | 8.9 | 12.6 | 18.2 | 34.0 |
| **Race/Ethnicity** |  |  |  |  |  |
| Hispanic White | 13.2 (14.8) | 8.4 | 11.5 | 16.1 | 28.1 |
| Non-Hispanic White | 13.3 (12.7) | 8.9 | 11.6 | 16.0 | 26.0 |
| African American | 16.6 (28.1) | 11.3 | 15.5 | 18.6 | 42.9 |
| Asian | 23.9 (29.5) | 8.6 | 18.3 | 30.2 | 46.9 |
| Others | 12.5 (16.0) | 8.1 | 10.7 | 14.7 | 27.9 |
| **Rhinitis in Last 12 Months** |  |  |  |  |  |
| None | 12.6 (13.0) | 8.1 | 11.1 | 14.9 | 24.5 |
| 1-12 months ago | 12.7 (11.8) | 9.4 | 11.4 | 15.9 | 25.0 |
| 7 Days - 1 month ago | 14.9 (16.7) | 9.8 | 12.2 | 18.0 | 33.9 |
| Within last 7 Days | 19.6 (31.7) | 10.3 | 15.3 | 25.1 | 49.2 |
| **Inhaled Corticosteroid Use in Last 12 Months** |  |  |  |  |  |
| Did not use | 13.2 (14.0) | 8.5 | 11.6 | 16.0 | 27.4 |
| Used 1-12 months ago | 19.3 (33.7) | 11.1 | 15.8 | 21.7 | 45.9 |
| Used within last 1 month | 36.3 (46.4) | 12.0 | 26.8 | 44.6 | 67.0 |
| **Asthma** |  |  |  |  |  |
| No | 12.6 (12.6) | 8.2 | 11.1 | 14.9 | 24.5 |
| Yes | 19.6 (32.7) | 11.0 | 16.1 | 25.4 | 51.2 |
| **Total** | 13.4 (14.6) | 8.6 | 11.6 | 16.5 | 28.4 |
