# Supplementary material for: Determinants of Children's Exhaled Nitric Oxide: New Insights from Quantile Regression
Source: PLoS One. 2015 Jul 27;10(7):e0130505. doi: 10.1371/journal.pone.0130505 (PMC4516246; doi:10.1371/journal.pone.0130505)
Supplement: S2 Table — (DOCX) [file pone.0130505.s002.docx]

**S2 Table: Pairwise Tests of the Equality of Covariate Effects across Selected Quantiles**

|  | **0.2 vs 0.5** | **0.2 vs 0.8** | **0.5 vs 0.8** |
| --- | --- | --- | --- |
| **Asthma** |  |  |  |
| Yes | 0.001 | <0.001 | <0.001 |
| **Rhinitis in Last 12 Months** |  |  |  |
| 1-12 months ago | 0.309 | 0.620 | 0.860 |
| 7 Days - 1 month ago | 0.300 | 0.159 | 0.258 |
| Within last 7 Days | 0.006 | <0.001 | 0.005 |
| **Gender** |  |  |  |
| Male | 0.005 | <0.001 | <0.001 |
| **Race** |  |  |  |
| Non-Hispanic White | 0.733 | 0.119 | 0.113 |
| African American | 0.893 | 0.840 | 0.818 |
| Asian | <0.001 | <0.001 | 0.631 |
| Others | 0.792 | 0.710 | 0.624 |
